# Influence of Agro-Industrial By-Products on *Tenebrio molitor* Biomass and Its Bench-Scale Fractionation by Supercritical CO_2_

**DOI:** 10.3390/molecules31213840

**Published:** 2026-10-24

**Authors:** Vincenzo Larocca, Marco Iannaccone, Maria Martino, Stefania Moliterni, Simona Errico, Raffaella Rebuzzi, Gian Paolo Leone, Anna Sabatino, Nicola Fedele, Patrizia Casella, Antonio Molino

**Affiliations:** 1 Trisaia Research Center, ENEA-Italian National Agency for New Technologies, Energy and Sustainable Economic Development, 75026 Rotondella, Italy; vincenzo.larocca@enea.it (V.L.); maria.martino@enea.it (M.M.); stefania.moliterni@enea.it (S.M.); simona.errico@enea.it (S.E.); raffaella.rebuzzi@enea.it (R.R.); 2 Portici Research Center, ENEA-Italian National Agency for New Technologies, Energy and Sustainable Economic Development, Piazzale Enrico Fermi 1, 80055 Portici, Italy; anna.sabatino@enea.it (A.S.); nicola.fedele@enea.it (N.F.); patrizia.casella@enea.it (P.C.); antonio.molino@enea.it (A.M.); 3 Casaccia Research Center, ENEA-Italian National Agency for New Technologies, Energy and Sustainable Economic Development, Via Anguillarese, 301, 00123 Roma, Italy; gianpaolo.leone@enea.it (G.P.L.)

**Keywords:** edible insects, supercritical CO_2_ extraction, protein enrichment, insect biorefinery

## Abstract

The integration of sustainable insect rearing with green downstream processing is essential for the development of circular biorefineries. This study investigated the fractionation of *Tenebrio molitor* biomass obtained from larvae reared on diets containing agro-industrial by-products by bench-scale supercritical CO_2_ (SC-CO_2_) extraction. Larvae were reared on diets containing brewer’s spent grain (BSG), cocoa bean shell (CBS), or banana peel (BP). A preliminary process screening identified 40 °C and 200 bar as the most suitable extraction conditions, providing efficient fractionation under mild operating conditions. Lipid extraction yield depended on the initial composition of the insect meals, ranging from 6.47 ± 0.26% for BSG-derived biomass to 23.02 ± 1.52% for CBS-derived biomass. SC-CO_2_ extraction simultaneously produced protein-enriched meals, increasing protein content up to 69.4 ± 0.7%, with protein enrichment ranging from 14.9% to 33.4%. The recovered oils exhibited a favorable fatty acid profile, with more than 72% unsaturated fatty acids, predominantly oleic and linoleic acids. These findings show that differences in the composition of insect biomass obtained under different rearing conditions are reflected in the yield and characteristics of the fractions recovered under identical extraction conditions. Integrating rearing substrate selection with SC-CO_2_ extraction represents an effective strategy to produce high-value protein-enriched meals and oils from agro-industrial by-products, supporting the development of sustainable insect biorefineries.

## 1. Introduction

The transition towards more sustainable food production systems has become one of the major scientific and technological challenges of the last decade. Continuous population growth, increasing demand for high-quality proteins, depletion of natural resources and the environmental impact of conventional livestock production are driving the search for alternative food sources capable of combining nutritional quality with reduced ecological footprints. Current projections indicate that global food production will need to increase substantially by 2050, while simultaneously reducing greenhouse gas emissions, land occupation and freshwater consumption. In this context, the development of circular bioeconomy strategies based on the valorization of renewable biological resources and agro-industrial side streams has attracted increasing attention as a promising approach to reconcile food production with environmental sustainability [1,2,3]. The concept of circular bioeconomy is particularly relevant because it promotes the conversion of underutilized biomass into value-added products while reducing waste generation and improving resource efficiency, thereby creating new opportunities for sustainable food production systems.

Among the various alternative protein sources currently under investigation, edible insects have emerged as one of the most promising solutions because of their favorable nutritional composition, efficient feed conversion, rapid life cycle and limited environmental impact. Compared with conventional livestock, insects require considerably lower amounts of land, water and feed while producing fewer greenhouse gas emissions. Moreover, many insect species can efficiently convert organic residues and agro-industrial by-products into protein-rich biomass, making them particularly suitable for integration into circular production systems [4,5]. Recent reviews have further highlighted that edible insects should no longer be regarded solely as alternative protein sources but rather as multifunctional biological platforms capable of simultaneously generating proteins, lipids, bioactive compounds and organic fertilizers within integrated biorefinery concepts [3,6].

Among edible insect species, *Tenebrio molitor* has received particular attention because of its positive nutritional profile and its established regulatory status within the European Union. Following the positive safety assessment published by the European Food Safety Authority (EFSA), yellow mealworm has become one of the first insect species authorized as a novel food, stimulating considerable interest in both academic research and industrial production [7]. Mealworm larvae typically contain high concentrations of proteins and lipids, characterized by a balanced amino acid profile and a fatty acid composition dominated by unsaturated fatty acids, particularly oleic and linoleic acids [3,8]. Furthermore, several studies have demonstrated that the nutritional quality of *T. molitor* can be significantly influenced by biological factors, processing conditions and, most importantly, diet composition, providing opportunities to tailor insect biomass according to specific nutritional or technological requirements [9,10,11,12,13,14].

The possibility of rearing mealworms on agro-industrial by-products has become one of the most active research areas within insect biotechnology. Numerous lignocellulosic residues and food-processing by-products, including brewer’s spent grain, fruit residues, cereal by-products and cocoa processing wastes, have been successfully evaluated as alternative feeding substrates. Besides reducing production costs, these materials represent abundant secondary resources that can be reintroduced into the food chain through insect bioconversion, contributing to waste reduction and improving the sustainability of the production system [9,10,11,12]. Several authors have reported that diet formulation influences not only larval growth and feed conversion efficiency but also protein, lipid and fatty acid composition, confirming that insect biomass can be nutritionally modulated through appropriate substrate selection [8,13,15]. Consequently, feed formulation should not simply be regarded as a production variable but as a technological strategy capable of generating insect biomasses with different compositional characteristics and potential downstream applications.

Parallel to the development of sustainable insect production, increasing attention has been devoted to environmentally friendly technologies for biomass fractionation. Conventional lipid extraction from insects relies on organic solvents such as hexane, which raise concerns related to solvent residues, environmental impact and process sustainability. In contrast, supercritical carbon dioxide (SC-CO_2_) extraction has emerged as one of the most promising green extraction technologies because carbon dioxide is non-toxic, non-flammable, inexpensive, readily recyclable and easily removed from the final products. Furthermore, SC-CO_2_ operates under relatively mild temperatures, reducing thermal degradation while simultaneously producing solvent-free extracts with high selectivity and quality [16,17]. However, SC-CO_2_ extraction also presents some limitations, including the need for high-pressure equipment, the energy requirements associated with CO_2_ compression, and the relatively low extraction efficiency for highly polar compounds [18]. The adoption of green extraction technologies is increasingly recognised as an essential component of sustainable food processing, since extraction often represents one of the most energy and solvent intensive operations within food biorefineries.

Several studies have successfully applied SC-CO_2_ extraction to edible insect biomass, demonstrating its ability to recover lipid fractions while simultaneously producing protein-enriched defatted meals. Laroche et al. [19] compared conventional and sustainable lipid extraction methods for edible insect meal and showed that SC-CO_2_ extraction can provide effective lipid recovery while preserving the functional properties of the resulting protein fraction. Purschke et al. [15] specifically investigated the pilot-scale SC-CO_2_ extraction of oil from *T. molitor* larvae, evaluating the effects of extraction conditions on extraction kinetics, defatting performance and the composition of the recovered oil. More recently, Fornari et al. [20] demonstrated that the physicochemical characteristics of insect biomass, particularly its lipid content, can influence the efficiency of SC-CO_2_ defatting. Salgado-Ramos et al. [21] further investigated *T. molitor* oil produced by SC-CO_2_, including process scale-up and sustainability aspects. Collectively, these studies have established the feasibility of SC-CO_2_ extraction for insect biomass and have primarily investigated the effects of extraction conditions, process configuration or intrinsic biomass characteristics on lipid recovery and product quality [15,19,20,21].

In parallel, the use of agro-industrial by-products for *T. molitor* rearing has been extensively investigated. Papastavropoulou et al. [9] demonstrated that feeding strategies can affect the growth and nutritional profile of *T. molitor* larvae, while van Broekhoven et al. [10] vestigated the growth performance and feed conversion efficiency of mealworm species reared on diets composed of organic by-products. Rumbos et al. [11] evaluated seed-cleaning process by-products as alternative substrates for *T. molitor* and *Alphitobius diaperinus*, whereas Serra et al. [12] recently demonstrated that different agro-industrial by-product diets can affect both growth performance and the nutritional value of *T. molitor* larvae. These studies clearly demonstrate that rearing substrate selection can generate insect biomasses with different biological and nutritional characteristics [9,10,11,12]. However, the downstream processing of these diet-derived biomasses has generally not been investigated in conjunction with the rearing stage. In particular, the effect of feed-driven differences in *T. molitor* biomass composition on subsequent SC-CO_2_ fractionation remains insufficiently explored.

Thus, in the present study the experimental integration of upstream rearing substrate selection with downstream SC-CO_2_ fractionation under identical extraction conditions is been investigated. *T. molitor* larvae were reared on three agro-industrial by-products, namely brewer’s spent grain (BSG), cocoa bean shells (CBS), and banana peels (BP), generating biomasses with markedly different chemical compositions. After a preliminary screening of SC-CO_2_ operating conditions, the selected conditions were applied without modification to the insect meals obtained from the different diets, allowing the effect of biomass composition to be evaluated independently of changes in extraction parameters. Lipid extraction yield, protein enrichment of the exhausted meals and the fatty acid composition of the recovered oils were subsequently compared. This approach moves beyond the separate optimisation of insect rearing and extraction and provides experimental evidence that feed-driven differences in insect biomass can be reflected in downstream fractionation performance, thereby supporting the development of integrated insect biorefineries in which upstream biomass production and downstream valorisation are considered interconnected stages.

## 2. Results

### 2.1. Development of the Bench-Scale Supercritical CO_2_ Extraction Process and Selection of the Operating Conditions

A preliminary experimental screening was carried out to identify the most suitable operating conditions for the bench-scale supercritical CO_2_ fractionation of *T. molitor* larvae meal. The screening was performed using meal obtained from larvae reared on the control diet, allowing the extraction process to be optimized before its application to biomasses produced from alternative agro-industrial by-products. Six operating conditions were investigated by combining two extraction pressures (200 and 300 bar) with three temperatures (40, 50 and 60 °C), while biomass loading (2 g), extraction time (95 min) and CO_2_ flow rate (4 L min^−1^) were kept constant.

**Figure 1:**
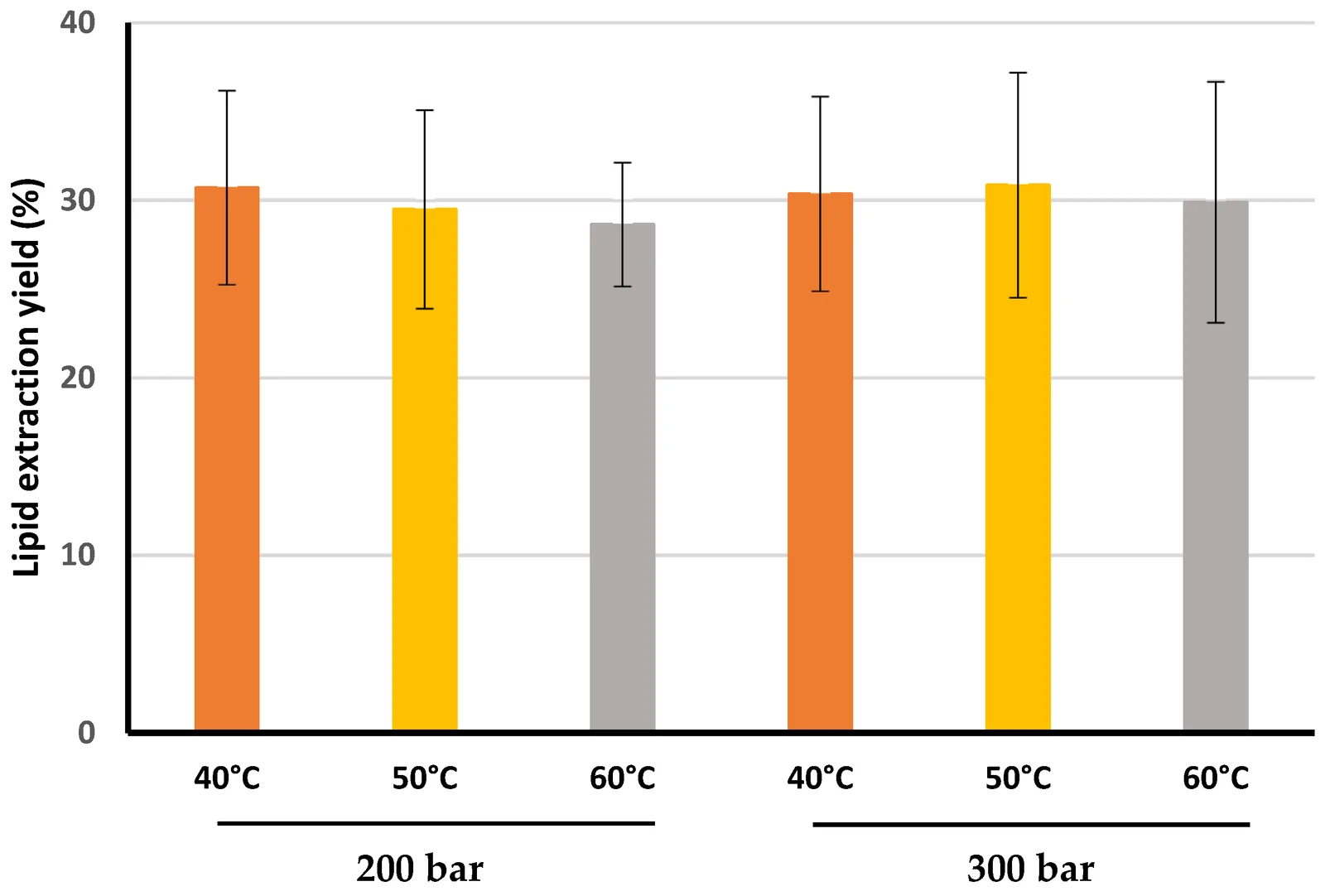
Effect of extraction pressure (200 and 300 bar) and temperature (40, 50 and 60 °C) on lipid extraction yield obtained by bench-scale supercritical CO_2_ extraction of *Tenebrio molitor* larvae meal. Biomass loading (2 g), extraction time (95 min) and CO_2_ flow rate (4 L min^−1^) were kept constant in all experiments. Values are expressed as mean ± standard deviation (number = 3). Statistical analysis was performed by one-way ANOVA. No significant differences were observed among the investigated temperature and pressure conditions (*p* > 0.05).

As shown in Figure 1, lipid extraction yield remained relatively stable across the investigated operating range, varying from 28.63 ± 3.49 to 30.85 ± 6.34% (dry basis).

As shown in Figure 1, lipid extraction yield showed only limited variation across the investigated pressure and temperature conditions, with no significant differences among the different temperatures and pressure investigated. At 200 bar, extraction yields were 30.70 ± 5.47, 29.49 ± 5.59 and 28.63 ± 3.49% at 40, 50 and 60 °C, respectively, while corresponding values at 300 bar were 30.35 ± 5.49, 30.85 ± 6.34 and 29.89 ± 6.78%. Although relatively high variability was observed among replicate extractions within some of the investigated conditions, this variability is compatible with that reported for SC-CO_2_ extraction of *T. molitor* flour and may be partly related to the intrinsic heterogeneity of the insect matrix, including lipid distribution and particle packing [22]. Within the investigated experimental domain, neither extraction pressure nor temperature significantly affected lipid recovery. These results suggest that efficient solubilization of the lipid fraction was already achieved under the mildest conditions evaluated, with no measurable advantage gained by increasing process severity. The fatty acid profiles obtained under the different extraction conditions are also reported in Supplementary Table S1.

Since the objective of the process was the integrated fractionation of insect biomass rather than the recovery of oil alone, the composition of the exhausted meals was also considered during process evaluation (Table 1).

Following supercritical CO_2_ extraction, all exhausted meals exhibited high protein concentrations, ranging from 66.6 ± 2.69% to 68.7 ± 2.43%, corresponding to protein enrichments of 40.2–44.6% relative to the untreated insect meal (reference value 47.5%). Residual lipids were below the limit of detection in all samples. No significant differences were observed in protein concentration among the six extraction conditions, indicating that variations in pressure and temperature within the investigated range did not significantly affect the composition of the exhausted meals.

### 2.2. Application of the Selected Operating Conditions Using Meals Obtained from Different Rearing Substrates

The operating conditions selected during the preliminary screening (40 °C and 200 bar) were subsequently applied to *T. molitor* meals obtained from larvae reared on three different agro-industrial by-products, BSG, CBSand BP. This experimental approach was adopted to compare the extraction performance and the composition of the resulting fractions from insect meals obtained from different rearing diets under identical operating conditions. The proximate composition of the insect meals is reported in Table 2.

As expected, the different feeding substrates produced meals with markedly different nutritional compositions. The BSG-derived meal exhibited the highest protein content (60.4% DM) and the lowest lipid content (12.6% DM), whereas the CBS-derived meal showed the lowest protein concentration (43.4%DM) and the highest lipid content (29.3% DM). The BP-derived meal displayed intermediate values, containing 50.9% protein and 24.8% lipids (DM).

The extraction performance obtained under the selected operating conditions is summarized in Table 3.

Lipid extraction yield differed significantly among the three insect meals, ranging from 6.47 ± 0.26% for the BSG-derived meal to 23.02 ± 1.52% for the CBS-derived meal, while the BP-derived meal yielded 19.43 ± 0.16%. The lipid extraction yield followed the same overall trend as the initial lipid content of the insect meals, with the CBS-derived meal showing the highest extraction yield, followed by the BP- and BSG-derived meals. This trend suggests that the greater lipid availability in the starting biomass contributed to the higher amount of lipid recovered under the same SC-CO_2_ operating conditions. The difference was particularly evident between the BSG- and CBS-derived meals, whereas the BP-derived meal showed an intermediate extraction yield, consistent with its intermediate initial lipid content.

The removal of lipids resulted in a corresponding increase in the protein concentration of the exhausted meals. Protein content increased to 69.4 ± 0.7% in the BSG-derived meal, 57.9 ± 1.4% in the CBS-derived meal and 63.2 ± 0.6% in the BP-derived meal, corresponding to protein enrichments of 14.9, 33.4 and 24.2%, respectively. The greater enrichment observed for the CBS-derived meal is consistent with its higher initial lipid content, as the removal of a larger lipid fraction proportionally increased the relative concentration of proteins in the exhausted biomass.

These results demonstrate that the selected operating conditions can be successfully applied to insect meals produced from different rearing substrates without requiring process modifications. Although the extraction yield depended on the initial chemical composition of the biomass, particularly its lipid content, the extraction process remained effective under identical operating conditions, confirming that its performance is primarily determined by the characteristics of the starting material rather than by the extraction protocol itself.

### 2.3. Fatty Acid Composition of the Extracted Oils

The fatty acid composition of the oils recovered by bench-scale supercritical CO_2_ extraction was determined to evaluate the effect of the different rearing substrates on the fatty acids composition of the lipid fraction. The complete fatty acid profiles of the extracted oils are reported in Table 4.

Regardless of the rearing substrate, all extracted oils were characterized by a predominance of unsaturated fatty acids, which accounted for more than 72% of the total identified fatty acid methyl esters (FAMEs). Monounsaturated fatty acids (MUFAs) represented the major lipid class, ranging from 40.61 to 48.08% of total FAMEs, followed by polyunsaturated fatty acids (PUFAs), which accounted for 24.44–35.66%. Saturated fatty acids (SFAs) constituted the smallest fraction of the lipid profile, representing between 23.73 and 27.48% of the total fatty acids. These results indicate that supercritical CO_2_ extraction efficiently recovered an oil characterized by a high proportion of nutritionally desirable unsaturated fatty acids.

Among the individual fatty acids, oleic acid (C18:1 n-9) was the predominant component in all samples, followed by linoleic acid (C18:2 n-6) and palmitic acid (C16:0). These three fatty acids accounted for the vast majority of the total lipid fraction, whereas all remaining fatty acids were detected only in minor amounts. The predominance of oleic and linoleic acids confirms the characteristic fatty acid profile reported for *T. molitor* larvae and highlights the nutritional value of the extracted oils [3].

The fatty acid profiles of the oils obtained from larvae reared on brewer’s spent grain and banana peel were remarkably similar, exhibiting almost identical proportions of SFAs, MUFAs and PUFAs. In contrast, the oil extracted from larvae reared on cocoa bean shell showed some differences in fatty acid distribution, characterized by a higher proportion of MUFAs, mainly due to the increased oleic acid content, and a corresponding reduction in PUFAs, primarily associated with a lower linoleic acid content. Despite these compositional differences, the fatty acid pattern remained comparable among the three oils, with unsaturated fatty acids consistently representing the predominant lipid fraction.

These findings demonstrate that the developed bench-scale supercritical CO_2_ extraction process consistently produced oils with a favorable fatty acid composition irrespective of the rearing substrate.

## 3. Discussion

The present study provides evidence that the composition of Tenebrio molitor biomass, as affected by the rearing substrate, can influence its subsequent behaviour during supercritical CO_2_ (SC-CO_2_) fractionation. While previous studies have mainly considered insect rearing [9,10] and downstream extraction as separate stages [19,22], the present approach connects these two processes by maintaining the extraction conditions constant and using insect meals with intentionally different compositions. This experimental design makes it possible to distinguish the effect of the starting biomass from that of the extraction parameters. The results therefore support the concept that feed formulation should be considered not only as a strategy to optimize insect growth and nutritional quality, but also as a means of producing biomasses with specific characteristics for subsequent valorisation. The influence of diet on the nutritional composition of edible insects has been extensively documented. Previous studies demonstrated that the incorporation of agro-industrial by-products into mealworm diets modifies larval growth, nutrient utilisation and the chemical composition of the resulting biomass, while maintaining its suitability for food and feed applications [1,8]. Similarly, recent reviews have highlighted that insect nutritional quality can be tailored through substrate selection, supporting the concept that feed formulation should be regarded as a technological tool rather than merely a production input [5,23]. The present findings extend this concept to downstream processing. In particular, the differences in lipid content generated by the rearing substrates were reflected in the amount of lipid recovered during SC-CO_2_ extraction. This relationship is consistent with the principle that the composition of the starting matrix represents an important factor governing supercritical extraction, in addition to pressure, temperature and other process variables [22]. Most studies investigating SC-CO_2_ extraction of edible insects have instead considered the insect meal as a fixed raw material, focusing on the optimisation of extraction variables such as pressure, temperature, extraction time and CO_2_ consumption. Supercritical defatting of *Hermetia illucens* demonstrated that extraction efficiency depends primarily on the physicochemical characteristics of the biomass, particularly lipid content, while moisture exerts a comparatively limited effect [20]. Likewise, Laroche et al. reported that SC-CO_2_ extraction of *T. molitor* achieved lipid extraction yields comparable to those obtained with conventional organic solvents while preserving the quality of both the extracted oil and the protein-rich residue [19]. More recently, optimisation studies have demonstrated that extraction yield can be improved through modifications of pressure, particle size distribution and extraction strategy, achieving defatting efficiencies exceeding 90% under laboratory conditions and approximately 84% after pilot-scale scale-up [24]. In contrast, the present study intentionally maintained all extraction variables constant, allowing the influence of biomass composition to be isolated from that of process conditions. The higher extraction yields observed for the CBS- and BP-derived meals, which also had higher initial lipid contents, are consistent with a greater amount of extractable lipid being available for solubilisation in the supercritical phase under the selected conditions. It should be noted that this relationship was observed at the fixed extraction time of 95 min, as no kinetic tests were performed; therefore, this interpretation applies specifically to the experimental conditions investigated. Conversely, the lower lipid content of the BSG-derived meal was associated with the lowest extraction yield. This experimental approach shows that differences in biomass composition generated during insect rearing can be reflected in SC-CO_2_ extraction yield even when the extraction protocol remains unchanged.

The nutritional quality of the recovered oil deserves further consideration because the fatty acid profile remained remarkably stable despite the different rearing substrates. Oleic and linoleic acids were the predominant fatty acids in the recovered oils, indicating that modifying the diet did not substantially alter the main characteristics of the lipid fraction. Similar fatty acid distributions have consistently been reported for *T. molitor* oils extracted using supercritical CO_2_, where oleic and linoleic acids are recognised as the predominant fatty acids independently of extraction scale. Comparable compositions have also been described after conventional extraction procedures, confirming that these fatty acids represent intrinsic characteristics of mealworm lipids rather than an effect of the extraction technology [25,26]. These observations indicate that SC-CO_2_ extraction efficiently removes lipids while preserving the favorable fatty acid profile of the recovered oils, supporting its application for the production of insect oil fractions.

The predominance of oleic acid is particularly relevant because this monounsaturated fatty acid has been associated with several beneficial physiological effects, including the modulation of inflammatory pathways, antioxidant responses and cell signalling mechanisms [27]. Likewise, increasing evidence supports the role of dietary linoleic acid in maintaining cardiovascular and metabolic health when consumed within balanced dietary patterns [28,29]. Although the present work was not designed to evaluate biological activity, the fatty acid composition obtained confirms that the recovered oil represents a valuable co-product rather than a simple by-product of the defatting process.

Besides improving protein concentration, solvent-free extraction offers important technological advantages over conventional lipid extraction methods. Previous comparisons between SC-CO_2_ and organic solvent extraction demonstrated that supercritical processing produces oils and protein fractions of comparable quality while eliminating solvent residues and reducing the environmental concerns associated with hexane extraction [19]. Similar conclusions were reached by Bolat et al., who reported that alternative defatting technologies significantly influence the technological properties of insect powders, emphasising the importance of selecting extraction methods according to the intended food application [30]. Furthermore, Queiroz et al. [8] highlighted that protein functionality is strongly affected not only by insect species but also by processing technologies, including drying and lipid removal, underlining the importance of selecting mild extraction conditions for preserving protein quality.

An additional aspect emerging from the present study concerns the choice of extraction conditions. The final process was intentionally performed at 40 °C and 200 bar, following the preliminary optimization study, because these conditions did not result in a measurable loss of extraction yield compared with the other conditions tested. Although several studies have reported higher defatting efficiencies using pressures above 300–400 bar, increasing pressure inevitably increases energy demand and compression costs, particularly during industrial scale-up [31,32]. Consequently, optimization of SC-CO_2_ extraction should not exclusively target maximum oil recovery but also the energy and economic implications of the process that was beyond the scope of the present study. This concept is consistent with recent reviews on green extraction technologies, which emphasize that extraction efficiency, energy consumption and product quality should be evaluated simultaneously when developing sustainable industrial processes [16,17].

The results obtained in the present work support a broader interpretation of insect biorefineries. Rather than considering insect production and downstream fractionation as independent operations, the data indicate that both stages should be integrated into a single process in which feed formulation contributes to the characteristics of the biomass entering downstream processing. Agro-industrial by-products therefore assume a dual function: they reduce dependence on conventional feed ingredients while simultaneously generating insect biomasses with distinct physicochemical characteristics that are reflected in downstream processing outcomes. This concept is fully consistent with recent biorefinery strategies proposing edible insects as platforms for the simultaneous production of proteins, lipids and other high-value compounds within circular bioeconomy models [2,6]. Indeed, recent reviews have recognized edible insects as one of the most promising alternative protein sources because of their ability to convert low-value biomass into nutritionally valuable food ingredients while reducing environmental burdens associated with conventional livestock production [3,4].

The present study indicates that the yield and characteristics of the fractions obtained by supercritical CO_2_ extraction are related not only to the extraction conditions but also to the biochemical characteristics of the insect biomass. By maintaining the extraction parameters constant, the study highlights how differences in the starting biomass, particularly in lipid content, are reflected in oil recovery and protein enrichment. Integrating feed formulation with green extraction technologies therefore represents a promising approach for the valorisation of *T. molitor* biomass within insect biorefineries. Future research should investigate the relationships between diet composition, biomass microstructure and extraction kinetics to develop predictive models capable of designing insect biomasses specifically tailored for sustainable downstream processing.

## 4. Materials and Methods

### 4.1. Production of Tenebrio Molitor Biomass

*T. molitor* larvae were reared at the ENEA laboratories (Trisaia Research Centre, Rotondella, Italy) under controlled environmental conditions (27 ± 1 °C and 65 ± 5% relative humidity) in continuous darkness. Four experimental diets were formulated, including a control diet composed of wheat bran (WB) and brewer’s yeast (ZY), and three diets in which wheat bran was partially replaced with brewer’s spent grain (BSG), cocoa bean shell (CBS), or banana peels (BP). The proximate composition of the individual feed ingredients is reported in Table 5, whereas the complete formulations of the experimental diets are provided in Table S2 (Supplementary Table S2).

The experimental diets were formulated to provide an identical crude protein content (16.6%, dry basis), while differing in the type and proportion of the agro-industrial by-products used as feed ingredients. Larvae were fed *ad libitum* throughout the 28 day experimental period, and fresh *Opuntia ficus-indica* cladodes were periodically supplied as a moisture source [33].

Approximately two-month-old larvae with a homogeneous initial body weight were randomly assigned to experimental containers (20 larvae per container), with ten biological replicates for each dietary treatment. At the end of the feeding trial, larvae were separated from the residual substrate by sieving and subjected to a 24 h starvation period to allow gut clearance prior to processing.

The harvested larvae were subsequently inactivated by freezing, freeze-dried to constant weight, and milled using a commercial blender (HGB55E, Model 38BL45, Waring Commercial, Torrington, CT, USA) to obtain a homogeneous insect meal. The resulting meals were stored at 4 °C in sealed containers protected from light until use in the subsequent bench-scale supercritical CO_2_ extraction experiments. Throughout this study, insect meals obtained from larvae reared on the control, BSG, CBS, and BP diets are referred to as Control, BSG, CBS, and BP meals, respectively.

### 4.2. Bench-Scale Supercritical CO_2_ Extraction

Supercritical CO_2_ extraction experiments were performed using a laboratory-scale supercritical fluid extraction system (Spe-ed SFE-2, Applied Separations, Allentown, PA, USA) installed at the ENEA Trisaia Research Centre (Rotondella, Italy). The extraction unit consisted of a liquid CO_2_ delivery system, a pneumatic high-pressure pump, a thermostatically controlled 50 mL stainless-steel extraction vessel, a back-pressure regulator (BPR), and a collection vessel for the recovery of the extracted lipids. Prior to compression, liquid CO_2_ was cooled using a recirculating refrigeration system (RW-1040G, Lab Companion, Republic of Korea) maintained at −2 °C. During extraction, pressure, temperature, and CO_2_ flow rate were continuously monitored and controlled using EasyCom2011 software (version 2.0.5.16, WIKA Alexander Wiegand SE & Co. KG). Following depressurization, the lipid extract was collected in the separator, whereas the gaseous CO_2_ flow was measured using a gas flow meter (LPN/S80 ALG 2.5, Sacofgas, Italy). The extracted oil and the corresponding defatted insect meal were collected separately and stored at −20 °C until further chemical characterization.

### 4.3. Experimental Design

A preliminary screening was carried out to identify suitable operating conditions for the supercritical CO_2_ extraction of *T. molitor* meals. Larvae from the individual rearing containers were pooled within each dietary treatment and subsequently freeze-dried and milled. Before each extraction, 2.0 g of the resulting insect meal were loaded into the extraction vessel. Six extraction conditions were evaluated by combining two pressure levels (200 and 300 bar) with three extraction temperatures (40, 50 and 60 °C), while extraction time (95 min) and CO_2_ flow rate (4 L min^−1^, expressed as the volumetric flow rate in the gaseous state) were kept constant throughout the experiments. All extractions were performed in triplicates. The investigated operating conditions are summarized in Table 6.

The lipid extraction yield was used as the primary response variable for the preliminary screening of the operating conditions. Based on these results, the most suitable extraction conditions were selected for the subsequent characterization of the meals obtained from larvae reared with the different experimental diets.

The extraction yield was calculated as the percentage ratio between the mass of the lipid extract recovered after supercritical CO_2_ extraction and the initial mass of freeze-dried insect meal loaded into the extraction vessel, according to the following equation (Equation (1))

(1) $$\mathrm{Lipid}\mathrm{extraction yield}\left(\%\right)=\frac{m_{\mathrm{extract}}}{m_{\mathrm{meal}}}\times 100$$

where *m_extract_* is the mass (g) of the lipid extract recovered after supercritical CO_2_ extraction and *m_meal_* is the initial mass (g) of freeze-dried insect meal loaded into the extraction vessel.

### 4.4. Proximate Composition Analysis

The proximate composition of untreated and defatted *T. molitor* meals was determined by measuring moisture, ash, crude protein, total carbohydrates, and lipid contents. Moisture content was determined according to UNI EN ISO 712-1:2024 [34] by drying the samples in a ventilated oven at 105 °C until constant weight. Ash content was determined according to UNI EN ISO 2171:2023 [35] by incineration in a muffle furnace at 550 °C for at least 4 h. Crude protein content was determined according to UNI EN ISO 20483:2013 [36] using the Kjeldahl method. Approximately 0.5–1.0 g of sample were analysed, and protein content was calculated using a nitrogen-to-protein conversion factor of 4.75 [37]. Total carbohydrate content was determined using the phenol–sulphuric acid colorimetric method. Calibration curves were prepared using D(+)-glucose standard solutions at concentrations of 20, 40, 60, 80, 100, and 150 mg L^−1^, and absorbance was measured at 485 nm [38]. Total lipid content was determined by Accelerated Solvent Extraction (ASE) using n-hexane as extraction solvent at 50 °C and 100 bar. Approximately 2.0–2.5 g of sample were subjected to three consecutive extraction cycles to ensure complete lipid recovery [39].

### 4.5. Fatty Acids Analysis

The fatty acid composition of the lipid extracts obtained by supercritical CO_2_ extraction was determined after conversion of the fatty acids into their corresponding methyl esters (FAMEs). FAME preparation and identification were performed according to UNI EN ISO 12966-4 [40] using an Agilent 7820A gas chromatograph coupled with a flame ionization detector (GC-FID Agilent 7820A, Santa Clara, CA, USA) and equipped with a J&W DB-23 capillary column (30 m × 0.25 mm i.d., 0.25 µm film thickness; model 122-2332, Agilent, Santa Clara, CA, USA). The injector and detector temperatures were set at 250 °C, while the oven temperature was programmed from 150 °C to 240 °C at a ramp rate of 4 °C min^−1^. Nitrogen (purity > 99.999%) was used as the carrier gas at a linear velocity of 30 cm s^−1^. Tricosanoic acid (C23:0) was used as the internal standard for fatty acid quantification. Individual fatty acids were identified by comparing their retention times with those of a certified FAME standard mixture. The relative abundance of each fatty acid was expressed as the percentage of the total identified FAMEs. Saturated fatty acids (SFAs), monounsaturated fatty acids (MUFAs), and polyunsaturated fatty acids (PUFAs) were calculated as the sum of the corresponding individual fatty acids. Analysis was performed on pooled samples.

### 4.6. Statistical Analysis

Statistical analyses were performed using Jamovi software (version 2.7; https://www.jamovi.org). Data were analysed by one-way analysis of variance (ANOVA), and Tukey’s honestly significant difference (HSD) test was applied for post hoc multiple comparisons when significant differences were detected.

## 5. Conclusions

The present study showed that the use of agro-industrial by-products as rearing substrates resulted in *T. molitor* biomasses with different chemical compositions, which were reflected in the yield and characteristics of the fractions obtained by supercritical CO_2_ extraction under identical operating conditions. In particular, differences in lipid extraction yield were closely associated with the initial lipid content of the insect meals, while the process simultaneously generated protein-enriched meals and oils rich in unsaturated fatty acids. The results highlight the importance of considering the composition of the starting biomass when applying standardized SC-CO_2_ fractionation protocols. Thus, the integration of feed formulation with green downstream processing represents a promising approach for the valorisation of *T. molitor* biomass and for the development of circular insect biorefineries. A limitation of the present study is that the functional properties of the protein-enriched meals and the oxidative quality and stability of the recovered oils were not assessed. Further studies combining controlled dietary manipulation with detailed extraction kinetics and process assessment are needed to clarify the relationships between biomass composition and downstream fractionation performance.

## Figures and Tables

**Table 1:** Protein concentration and residual lipid content of the exhausted *Tenebrio molitor* meals obtained after bench-scale supercritical CO_2_ extraction under the six investigated operating conditions. Protein enrichment was calculated relative to the untreated insect meal (reference value 47.5%). Values are expressed as mean ± standard deviation (number = 3). Statistical analysis was performed by one-way ANOVA and no significant differences were observed among the investigated treatments (*p* > 0.05) LOD: limit of detection; SFE 1-SFE6: supercritical fluid extraction conditions are reported in the material and method section.

Sample	Protein (%)	Relative Protein Enrichment (%)	Lipid (%)
Exhausted SFE 1	68.6 ± 1.65	44.42	<LOD
Exhausted SFE 2	67.4 ± 2.39	41.89	<LOD
Exhausted SFE 3	66.6 ± 2.69	40.21	<LOD
Exhausted SFE 4	68.2 ± 2.82	43.58	<LOD
Exhausted SFE 5	68.7 ± 2.43	44.63	<LOD
Exhausted SFE 6	67.8 ± 2.64	42.74	<LOD

**Table 2:** Proximate composition of *Tenebrio molitor* meals obtained from larvae reared on different agro-industrial by-products before bench-scale supercritical CO_2_ extraction. Analyses were performed on representative pooled samples from each dietary treatment. Values are expressed as percentage on a dry matter basis (DM). BSG: brewer’s spent grain; CBS: cocoa bean shells; BP: banana peels.

	Crude Protein (DM%)	Carbohydrates (DM%)	Total Lipids (DM%)	Ashes (DM%)
Control diet	47.5	1.5	22.2	0.9
Diet1-BSG	60.4	2.7	12.6	1.0
Diet 2-CBS	43.4	2.6	29.3	0.7
Diet 3-BP	50.9	2.1	24.8	0.8

**Table 3:** Extraction yield and protein enrichment of *Tenebrio molitor* meals obtained after bench-scale supercritical CO_2_ extraction under the selected operating conditions (40 °C and 200 bar). Protein enrichment was calculated relative to the corresponding untreated insect meal. Values are expressed as mean ± standard deviation (n = 3). Different letters indicate significant differences among meals derived from larvae reared with different diets (one-way ANOVA followed by Tukey’s post hoc test, *p* < 0.05). BSG: brewer’s spent grain; CBS: cocoa bean shells; BP: banana peels.

Sample	Lipid Extraction Yield (%)	Protein in Exhausted Meal (%)	Relative Protein Enrichment (%)
Diet 1-BSG	6.47 ± 0.26 ^a^	69.4 ± 0.7	14.9
Diet 2-CBS	23.02 ± 1.52 ^b^	57.9 ± 1.4	33.4
Diet 3-BP	19.43 ± 0.16 ^c^	63.2 ± 0.6	24.2

**Table 4:** Fatty acid composition (% of total identified fatty acid methyl esters, FAMEs) of oils recovered by bench-scale supercritical CO_2_ extraction from *Tenebrio molitor* larvae reared with diet based on brewer’s spent grain (BSG), cocoa bean shells (CBS), and banana peels (BP). Oils obtained from each dietary treatment were pooled before analysis and values are expressed as percentage of total identified FAMEs. FAME: fatty acid methyl esters; SFA: saturated fatty acids; MUFA: monounsaturated fatty acids; PUFA: polyunsaturated fatty acids.

Fatty Acid (%)	Diet 1—BSG	Diet 2—CBS	Diet 3—BP
C12:0	0.28	0.30	0.28
C13:0	0.11	0.08	0.11
C14:0	3.95	3.87	3.98
C15:0	0.27	0.23	0.27
C16:0	15.64	17.64	15.74
C17:0	0.30	0.29	0.30
C18:0	3.18	5.07	3.15
Σ SFA	23.73	27.48	23.83
C14:1	<0.01	<0.01	<0.01
C16:1	2.66	2.75	2.68
C17:1	0.32	0.31	0.32
C18:1 n-9	37.63	45.02	37.63
Σ MUFA	40.61	48.08	40.62
C18:2 n-6	35.66	24.44	35.55
Σ PUFA	35.66	24.44	35.55

**Table 5:** Proximate composition of the feed ingredients used for the preparation of the experimental diets.

Feed Ingredient	Crude Protein (%)	Lipids (%)	Carbohydrates (%)	Moisture (%)	Ash (%)
Wheat bran (WB)	15.0	6.7	13.0	11.7	5.7
Brewer’s yeast (ZY)	46.0	0.33	13.4	8.2	3.3
Brewer’s spent grain (BSG)	18.0	5.0	11.4	6.6	5.2
Cocoa bean shell (CBS)	18.0	3.1	6.2	7.8	12.2
Banana peels (BP)	6.7	3.4	9.3	15.1	21.1

**Table 6:** Experimental conditions evaluated during the preliminary screening of bench-scale supercritical CO_2_ extraction of *Tenebrio molitor* meals.

Operating Condition				
Run	Temperature (°C)	Pressure (bar)	Duration Time (min)	Flow (L min^−1^)
SFE 1	40	200	95	4
SFE 2	50	200	95	4
SFE 3	60	200	95	4
SFE 4	40	300	95	4
SFE 5	50	300	95	4
SFE 6	60	300	95	4

## Data Availability

The datasets generated or analyzed during the current study are available from the corresponding author upon reasonable request.

## Supplementary Materials

The following supporting information can be downloaded at: https://www.mdpi.com/article/10.3390/molecules31213840/s1, Supplementary Table S1. Fatty acid profile of the lipid extracts obtained from *Tenebrio molitor* meal reared on the control diet under the six supercritical CO_2_ extraction conditions investigated during the preliminary screening. FAs/total lipids represents the percentage of identified fatty acids relative to total extracted lipids, while SFA, MUFA, and PUFA represent the relative proportions of saturated, monounsaturated, and polyunsaturated fatty acids, respectively. SFE-1: 40 °C-200 bar; SFE-2: 50 °C-200bar; SFE-3: 60 °C-200bar; SFE-4: 40 °C-300 bar; SFE-5: 50 °C-300 bar; SFE-6: 60 °C-300 bar. Supplementary Table S2. Composition of the diets used for *Tenebrio molitor* rearing. Values are expressed as percentages on a weight-to-weight basis (% *w*/*w*) of the diet. Control diet, Diet 1-BSG, Diet 2-CBS, and Diet 3-BP correspond to the wheat bran-based control diet and the diets formulated with brewer’s spent grain (BSG), cocoa bean shell (CBS), and banana peels (BP), respectively.
