# Supplementary material for: Influence of Agro-Industrial By-Products on Tenebrio molitor Biomass and Its Bench-Scale Fractionation by Supercritical CO2
Source: Molecules. 2026 Oct 24;31(21):3840. doi: 10.3390/molecules31213840 (PMC13765292; doi:10.3390/molecules31213840)
Supplement: Supplementary file 1 [file molecules-31-03840-s001.zip › molecules-4532317-supplementary.pdf]

Supplementary Table S1. Fatty acid profile of the lipid extracts obtained from *Tenebrio molitor* meal reared on the control diet under the six supercritical CO_2 extraction conditions investigated during the preliminary screening. FAs/total lipids represents the percentage of identified fatty acids relative to total extracted lipids, while SFA, MUFA, and PUFA represent the relative proportions of saturated, monounsaturated, and polyunsaturated fatty acids, respectively. SFE-1: 40°C-200 bar; SFE-2: 50°C-200bar; SFE-3: 60°C-200bar; SFE-4: 40°C-300 bar; SFE-5: 50°C-300 bar; SFE-6: 60°C-300 bar.

	**SFE-1**	**SFE-2**	**SFE-3**	**SFE-4**	**SFE-5**	**SFE-6**
**FAs/total lipids (%)**	37.85	82.10	43.73	84.60	60.32	47.71
**Σ SFAs(%)**	23.11	22.73	38.89	39.89	36.74	40.91
**Σ MUFAs (%)**	46.04	45.82	56.73	57.60	58.05	56.94
**Σ PUFAs (%)**	30.85	31.45	4.38	2.51	5.21	2.16

Supplementary Table S2. Composition of the diets used for *Tenebrio molitor* rearing. Values are expressed as percentages on a weight-to-weight basis (% w/w) of the diet. Control diet, Diet 1-BSG, Diet 2-CBS, and Diet 3-BP correspond to the wheat bran-based control diet and the diets formulated with brewer’s spent grain (BSG), cocoa bean shell (CBS), and banana peels (BP), respectively.

**Ingredient (% w/w)**	**Control diet**	**Diet 1-BSG**	**Diet 2-CBS**	**Diet 3-BP**
Wheat bran	95.0	16.7	16.7	18.1
Brewer's spent grain	–	63.2	–	12.9
Cocoa bean shell	–	–	63.2	–
Banana peels	–	16.7	16.7	51.6
Brewer's yeast	5.0	3.3	3.3	17.5
